# Herpes Simplex Virus Type 1 DNA is associated with lower cognitive performance across multiple domains in Alzheimer's disease cases

**DOI:** 10.1002/alz70855_106840

**Published:** 2025-12-25

**Authors:** Marlene Tejeda, John J. Farrell, Congcong Zhu, Lee Wetzler, Kathryn L. Lunetta, Jesse Mez, William S Bush, Eden R. Martin, Li‐San Wang, Gerald D. Schellenberg, Margaret Pericak‐Vance, Jonathan L Haines, Paul K Crane, Shubhabrata Mukherjee, Timothy J. Hohman, Lindsay A. Farrer, Richard Sherva

**Affiliations:** ^1^ Boston University School of Medicine, Boston, MA, USA; ^2^ Department of Medicine (Biomedical Genetics), Boston University Chobanian & Avedisian School of Medicine, Boston, MA, USA; ^3^ Boston University Chobanian & Avedisian School of Medicine, Boston, MA, USA; ^4^ Department of Biostatistics, Boston University School of Public Health, Boston, MA, USA; ^5^ Department of Population & Quantitative Health Sciences, Case Western Reserve University School of Medicine, Cleveland, OH, USA; ^6^ John P. Hussman Institute for Human Genomics, Miller School of Medicine, University of Miami, Miami, FL, USA; ^7^ Penn Neurodegeneration Genomics Center, Department of Pathology and Laboratory Medicine, University of Pennsylvania Perelman School of Medicine, Philadelphia, PA, USA; ^8^ Penn Neurodegeneration Genomics Center, Department of Pathology and Laboratory Medicine, Perelman School of Medicine, University of Pennsylvania, Philadelphia, PA, USA; ^9^ Dr. John T. Macdonald Foundation Department of Human Genetics, University of Miami Miller School of Medicine, Miami, FL, USA; ^10^ Case Western Reserve University School of Medicine, Department of Population & Quantitative Health Sciences, Cleveland Institute for Computational Biology, Cleveland, OH, USA; ^11^ Department of Population and Quantitative Health Sciences, Institute for Computational Biology, Case Western Reserve University, Cleveland, OH, USA; ^12^ University of Washington School of Medicine, Seattle, WA, USA; ^13^ Department of Medicine, University of Washington, Seattle, WA, USA; ^14^ Vanderbilt Memory & Alzheimer's Center and Vanderbilt Genetics Institute, Vanderbilt University Medical Center, Nashville, TN, USA; ^15^ Departments of Medicine (Biomedical Genetics), Neurology, Ophthalmology, Biostatistics, and Epidemiology, Boston University Schools of Medicine and Public Health, Boston, MA, USA; ^16^ Biomedical Genetics, Department of Medicine, Boston University Medical School, Boston, MA, USA

## Abstract

**Background:**

Several viruses, including HSV‐1, can establish latent infections in neurons and have been linked to Alzheimer disease (AD).

**Method:**

Whole genome sequence reads derived from brain (561 AD cases, 259 controls) and blood (5,033 AD cases, 4,907 controls) specimens obtained from European ancestry, African American, Native American Hispanic, Indian, and Caribbean Hispanic participants in the Alzheimer's Disease Sequencing Project that did not align to the human reference genome were aligned to viral reference genomes. We evaluated the association of HSV‐1 DNA presence with harmonized measures of cognitive performance in four cognitive domains (memory, executive functioning, language, and visual‐spatial perception) using regression models with covariates for AD diagnosis, *APOE* genotype, age, sex, tissue source, ancestry, and PCR amplification. One model evaluated the interaction between HSV‐1 and age on cognitive decline using Generalized Estimating Equations to account for repeated measures. A second model considered only the most recent cognitive scores. Both models were performed separately within AD cases, mild cognitively impaired (MCI), and controls.

**Result:**

In the cross‐sectional; model, HSV‐1 was associated with lower memory performance in AD cases (b= ‐0.082, *p* = 0.002). HSV‐1 was significantly associated with decline across multiple domains, including executive function (b = ‐0.032, *p* =  0.012), language (b = ‐0.028, *p* = 0.027), and memory (b = ‐0.033, *p* = 0.008). Among AD cases, HSV‐1 was associated with reduced memory scores (b= ‐0.10, *p* =  4.35x10^‐6^). The HSV‐1 × age interaction term was significant for executive function (b = ‐0.003, *p* = 0.02) and memory (b = ‐0.003, *p* = 0.02) domains. In individuals with MCI (b = 0.012, *p* = 0.046) and controls (b = 0.091, *p* = 0.016), HSV‐1 was associated with higher visual‐spatial scores.

**Conclusion:**

These findings provide further evidence for the role of HSV‐1 presence in AD pathogenesis and may exacerbate cognitive decline in individuals with AD.

